# Working Women in Large Towns

**Published:** 1890-11-22

**Authors:** 


### Working Women in Large Towns.

### XVI.—SOCIAL, MORAL, AND PHYSICAL ASPECTS OF THE SUBJECT.—(*Continued.*)

Our efforts to improve the quality of women's labour, by giving them more technical training, may be expected to do far more for them than merely raise their wages. High wages, as has often been remarked, do not necessarily produce comfort and prosperity,—though we may point out, by the way, that this only holds good where a temporary rise in wages is concerned, and that when once a permanent improvement in the condition of a certain class has been effected, an improvement in their morals, manners, and ways of living is sure to follow. But quite apart from the question of wages, technical training must of itself do much for our workers. It is the least skilled and the worst paid, among men and women alike, who are most given to early marriages and general recklessness; and this being so, we are confident that higher skill will bring with it higher standards of living, greater prudence and thrift—in a word, all the advantages which education of any kind is wont to bestow.

As to irregularity of employment, which is acknowledged on all hands to be very injurious, it would seem to be inseparable from many trades, though we are of opinion that something might be done, not only by employers, but by consumers also, to distribute the work more evenly over the year, and thus diminish the length of those terrible "slack seasons" which are the ruin of so many workgirls. At the same time, the girls should be encouraged to look ahead, and to make some provision for these slack seasons, the horror of which would then be greatly mitigated. We think also that employments which in their very nature are and must be irregular (such, for instance, as fruit picking at a jam factory), should be left as far as possible to married women, to whom irregular work is really more suitable than constant employment. Miss Collet remarks that "no girl who respects herself will be content with irregular work, if she can possibly obtain work elsewhere"; and this instinctive sense of its dangers should be fostered by all means in our power.

Other evils connected with women's labour, as it at present is, are small and stuffy workshops, long hours, and an excessive amount of standing, all of which have an injurious effect upon the physical health of the workers, and should be prevented by combination, the pressure of public opinion, or, if other means fail, by legislation. We must also mention the censurable negligence of some employers, who do not trouble themselves as to the morals of the girls they engage, or the treatment of the girls when there. There are many good and conscientious employers; but there are also many careless ones; and if an employer appoints a mean, tyrannical foreman, or one of immoral character, he cannot but be held responsible for the consequences which may ensue. In fairness to the employer, we must state that a terrible charge which has sometimes been brought against members of his class—that they make bargains based on the loss of character of their *employés*, has been inquired into both in England and in America, and that no evidence for it is forthcoming. But characters may be lost, and souls ruined, by negligence as well as of malice prepense; and all employers—aye, and all members of companies which employ women-workers—should be given, if need be, a rude awakening to the knowledge that the ruin of many a poor workgirl must be laid to their charge, if they treat her, or allow her to be treated, as a mere "hand," with no claim upon them except for her weekly wage, no soul to be lost or saved.

We cannot close this article without a brief reference to three important difficulties in the way of those who would help our working-girls; their improvidence, their early marriages, and their love of drink. When the latter is caused by the exhaustion consequent upon long hours and severe labour, we may feel that little can be done until their conditions of life have been altered; but apart from this, we must look to more direct efforts than have yet been spoken of for the social and moral improvement of working-girls. The love of drink—and we may add, the love of such dangerous places as dancing saloons, and low-class music-halls, displayed by some of these girls, is simply a craving for some excitement after the labour and monotony of the day. Their homes are dull; there is no safe provision for them in the streets, or the ordinary places of amusement; and unless a helping hand is stretched out to them, they will take to bad ways, just for lack of something better to do. An evening club, with its bright warm parlours, and varied interests, may be a perfect godsend to such a girl, weaning her from the love of drink, giving her a taste for simple and innocent pleasures, and, moreover, greatly tending to prevent (so authorities on the subject tell us) the early marriages which we all condemn and deplore. A workgirl's home is too often no home at all, in the true sense of the word. Where there is no other alternative, she sets up a home of her own with a boy-sweetheart, while she is but a child herself—sometimes even giving as her reason for so doing, "Well, he was out o' work, yer see." But with a club of her own to turn to, she becomes not merely contented and happy, but self-respecting and prudent. Here, too, are gradually instilled into her higher ideals of home and home-life; and with the desire of one day possessing a good home of her own may very likely come the providence which shall make this possible. In various ways, then, such a club will benefit her, and not her alone, but the next generation.

